# The effect of varying levels of vehicle automation on drivers’ lane changing behaviour

**DOI:** 10.1371/journal.pone.0192190

**Published:** 2018-02-21

**Authors:** Ruth Madigan, Tyron Louw, Natasha Merat

**Affiliations:** Institute for Transport Studies, University of Leeds, Leeds, United Kingdom; Chongqing University, CHINA

## Abstract

Much of the Human Factors research into vehicle automation has focused on driver responses to critical scenarios where a crash might occur. However, there is less knowledge about the effects of vehicle automation on drivers’ behaviour during non-critical take-over situations, such as driver-initiated lane-changing or overtaking. The current driving simulator study, conducted as part of the EC-funded AdaptIVe project, addresses this issue. It uses a within-subjects design to compare drivers’ lane-changing behaviour in conventional manual driving, partially automated driving (PAD) and conditionally automated driving (CAD). In PAD, drivers were required to re-take control from an automated driving system in order to overtake a slow moving vehicle, while in CAD, the driver used the indicator lever to initiate a system-performed overtaking manoeuvre. Results showed that while drivers’ acceptance of both the PAD and CAD systems was high, they generally preferred CAD. A comparison of overtaking positions showed that drivers initiated overtaking manoeuvres slightly later in PAD than in manual driving or CAD. In addition, when compared to conventional driving, drivers had higher deviations in lane positioning and speed, along with higher lateral accelerations during lane changes following PAD. These results indicate that even in situations which are not time-critical, drivers’ vehicle control after automation is degraded compared to conventional driving.

## Introduction

Advanced driver assistance systems (ADAS) are becoming increasingly accessible, with systems such as the Volvo IntelliSafe Autopilot [1], and the Tesla Model S Autopilot [2] currently providing vehicle automation at SAE Level 2 [3]. The next step in vehicle automation development will be the trial of vehicles operating at SAE Level 3, where the vehicle provides sustained lateral and longitudinal vehicle control, with the understanding that the driver will intervene when requested to do so [3]. Although this increased automation of the driving task has the potential to lead to safety benefits such as a reduced number of crashes [4], along with potentially reducing vehicle emissions [5], it will also result in a fundamental shift in the drivers’ role from that of an active participant to a passive supervisor [6, 7]. The impact of this role change is likely to lead to reduced situation awareness, or knowledge of what’s happening in the environment [8], and “out-of-the-loop” performance problems, which have been shown to impair drivers’ ability to assume manual vehicle control in a timely and appropriate manner [9–14]. The effects of the changing demands on drivers’ attention and involvement in the driving task are likely to vary depending on the level of automation, as defined by SAE [3].

Until recently, much of the research into the effects of automation has focused on drivers’ responses to critical situations where the automated system reaches a limitation, and a transfer of control back to the driver is required. The majority of these studies have used driving simulators to investigate the impact of automation on driver behaviour during the transition. Some of the most highly researched issues arising during these critical transitions of control include (i) response times to critical and imminent take-over requests [10, 15]; (ii) the pattern of drivers’ eye movements during the transition of control [12, 14], (iii) brake and steering patterns after retaking control [16, 17], and (iv) vehicle positioning and stabilisation in the moments after a takeover request [13, 18]. Results have shown that while drivers can respond quite quickly to these take-over requests, they are associated with costs in terms of vehicle control [10, 13]. For example, when compared to manual driving, results show that following resumption of control from automation, drivers exhibit sharper trajectories and increased levels of high frequency steering activity, along with increased lateral and longitudinal accelerations, and higher brake pedal inputs [10, 16, 19]. These effects are exacerbated when the driver engages in other, non-driving related tasks, while the automation is on [7].

Although there is mounting evidence to suggest that drivers’ performance suffers during system-initiated transfers of control, less is known about the quality of driver-initiated take-overs in non-urgent scenarios. With an increasing number of vehicles having functionality such as Adaptive Cruise Control (ACC) and Lane Keeping Assist (LKA) as standard, these driver-initiated transfers are likely to become more common, for example when drivers wish to change the vehicle’s trajectory to overtake a lead vehicle, or to exit a motorway. In these types of situations drivers have more control over the take-over process, and can take some time to regain situation awareness before resuming control. A recent paper by Eriksson & Stanton [20] showed that when drivers were given a takeover request without a time restriction, there was large variability across participants in the time taken to resume control. In particular, there was a significant increase in response time when drivers were engaged in a secondary task during automation—resumption times ranged from 1.97 s to 25.75 s. Engagement in a secondary task did not lead to any significant increase in corrective steering actions, as measured by the standard deviation of steering angular rate. However, there was no comparison between drivers’ vehicle control performance with an automated system and conventional, manual driving. Thus, more research is needed to gain a clearer understanding of whether there are any performance decrements associated with drivers’ vehicle control in these non-critical situations, and whether the effects vary in any way at different levels of automation. The current study addresses this issue by examining drivers’ behaviour during lane changes in manual driving, partially automated driving (PAD), and conditionally automated driving (CAD).

Changing lane represents a safety-relevant driving manoeuvre which incorporates many of the critical aspects of driving. These include basic vehicle control elements, such as smoothly steering from one lane to an adjacent lane, and higher-order perceptual elements, such as maintaining situation awareness, decision-making and decision-execution [21–23]. Problems when changing lane can have a negative impact on both traffic safety and traffic flow [24], with approximately 539,000 two-vehicle lane change crashes occurring in the U.S. in 1999 [25]. It is possible that having to re-take control from an automated system to initiate a lane change will increase this risk. Therefore, it is important to gain an understanding of the effects of automation on drivers’ overtaking performance.

Previous studies have developed models of drivers’ decision-making during lane change and overtaking manoeuvres, identifying a number of key issues which drivers need to consider. These include the choice of lane, gap acceptance, relative speed, distance to the vehicle ahead, and distance to the point at which a lane change must be completed (e.g. [26–29]). However, little is known about the effects of these factors on drivers’ experience of overtaking while using different levels of automation. A study by Abe, Sato, and Itoh [30] showed that drivers had different requirements for passing bicycles and scooters during automated driving compared to when they were in control of the vehicle. They reported higher levels of trust and comfort when a larger lateral distance and earlier steering timing was adopted in automation, even if this did not match their manual driving behaviour. However, the study only examined drivers’ subjective evaluations of the overtaking scenarios during automation, and drivers did not have any control over the overtaking manoeuvre itself.

### Current study

The aim of the current study was to consider the above issues, by examining drivers’ experiences and vehicle control while changing lanes in manual driving, partially automated driving (PAD), and conditionally automated driving (CAD). We looked at how, and when, drivers initiated an overtaking manoeuvre during manual driving, and compared this to when they were interacting with a PAD and CAD system. In PAD, drivers were required to resume manual control of the vehicle in order to make a lane change, while in CAD, the automated system controlled all aspects of the driving task including the lane change, but drivers used the indicator lever to initiate the manoeuvre.

In particular, the study sought to address the following questions:

1. Are there any differences between manual driving, PAD, and CAD, regarding the time at which drivers initiate an overtaking manoeuvre?
2. Are there any differences in the distance to a lead vehicle at which drivers overtake in manual, PAD, and CAD?
3. Are there any differences in drivers’ vehicle control, as measured by lateral and longitudinal accelerations and lateral positioning during the overtaking manoeuvre, when drivers are fully in control (manual), compared to when they are required to resume control from automation (PAD)?
4. Are there any differences in drivers’ subjective evaluation of PAD and CAD systems?

## Method

### Participants

Following approval from the University of Leeds Research Ethics Committee (Reference Number LTTRAN-054), 30 participants were recruited for the study. 1 participant dropped out, leaving a total of 29 participants who completed the experiment (15 male), with an age range of 21–60 years (M = 34.21 years, SD = 8.94). All participants held a full driving licence for a minimum of 2 years (M = 13.62 years, SD = 9.62) and were regular drivers, driving an average of 8092.00 miles per year (SD = 7151.28). Participants were recruited via the University of Leeds Driving Simulator database, and received a payment of £20 in appreciation of their time.

### Materials & design

The experiment took place in the University of Leeds Driving Simulator (UoLDS), which consists of a Jaguar S-type cab with all driver controls operational. The vehicle is housed in a 4 m spherical projection dome and has a 300° field of view projection system. A Seeing Machines faceLAB eye-tracker was used to record eye movements at a rate of 60Hz.

**Fig 1:**
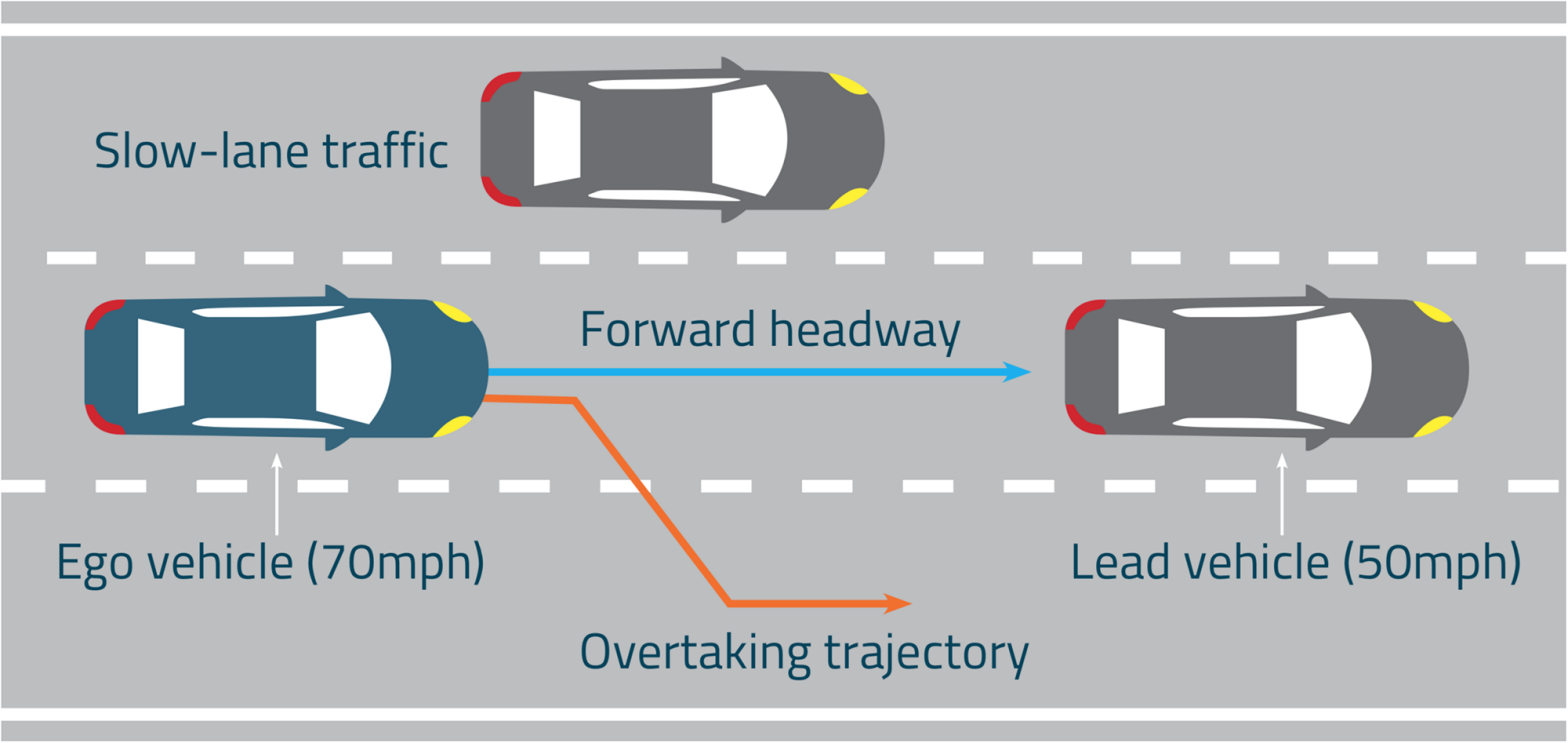
Representation of the traffic scenario for the lane change experiment.

All drives were completed on a three-lane motorway, which included straight and curved sections of road. It should be noted that this experiment was designed around a UK road, where vehicles travel on the left. There was a continuous stream of slow-moving traffic on the inside lane (left-hand lane) and no traffic in the outside lane (right-hand lane, see Fig 1). The speed limit was set at 70 mph, which is the national speed limit in the UK.

This study adopted a repeated-measures design with three drives:

1. A manual drive, where drivers had full control of the vehicle and were asked to overtake any vehicle travelling more slowly than them in the centre lane (SAE level 0).
2. A partially automated drive (PAD), operating at SAE Level 2, in which the automated system controlled driver speed, lane positioning, and distance to vehicles ahead (minimum forward headway of 2 s). However, drivers were required to disengage automation and resume manual control to overtake any slow moving lead vehicles. Vehicle automation could be disengaged by either pressing a button on the steering wheel, turning the steering wheel more than 2°, or pressing the brake or accelerator pedals. After completing an overtaking manoeuvre, drivers were required to re-engage the automation by pressing a button on the steering wheel.
3. A conditionally automated drive (CAD), operating at SAE Level 3, in which automation performed the vehicle control aspects of the driving task, including any overtaking manoeuvres. However, drivers had to use the indicator lever to initiate a lane change manoeuvre in either direction, and were required to monitor the system and the driving scene.

The order in which participants experienced each drive was counterbalanced.

For the manual drive, participants were asked to travel in the centre lane, and drive at the speed limit. For both the PAD and CAD drives, automation was only available when the driver was in the centre lane and travelling at a speed of approximately 70 mph. Drivers were instructed to engage automation as soon as possible at the start of both automated drives.

There were a total of 12 overtaking events in each drive, all initiated on straight segments of the road. For each of these events, a vehicle entered the driver’s lane from the slow lane (left lane in the UK), at a distance of approximately 180 m ahead of the driver, and travelled at a speed of 50 mph, approximately 20 mph slower than the driver’s vehicle (see ego vehicle in Fig 1). Each event ended once the driver had returned to the middle lane and re-engaged automation if required. There was a 30 second gap between each event.

### Procedure

On arrival at UoLDS participants were briefed about the experiment and filled out a consent form and initial questionnaire containing questions about their age, gender, mileage, etc. To assess whether participants’ behaviour was affected by their general attitudes towards automation, eight questions were administered using a seven-point anchored scale. All participants then completed a practice drive, accompanied by the experimenter, where they became accustomed to the simulator environment and vehicle controls. During the practice drive, they first drove manually for approximately 10 minutes and were encouraged to change lanes a number of times. Participants were then given the opportunity to practice the automated drive. They were asked to engage the automation by pressing a button on the steering wheel, after which they completed six overtaking manoeuvres. After the practice drive, participants completed the first experimental drive. This was followed by another short practice drive and the second and third experimental drives. At the start of each drive, they were reminded to overtake every slow moving lead vehicle, and to return to the centre lane once they had done so. Participants were allowed a short break after each drive, during which the next drive was set up in the simulator. Immediately after each of the PAD and CAD drives, they completed a questionnaire, which incorporated questions on system acceptance [31], the System Usability Scale [32], and a Human-Machine Interface (HMI) Evaluation Scale (adapted from [33]). At the end of the experiment, they completed a final questionnaire which included items on their preferred system, and a series of questions about their attitudes towards automation. Only the system acceptance and preferred system items are reported in this paper.

### Statistical analysis

Statistical analyses were completed using IBM SPSS v21. Shapiro Wilk’s tests showed that the data for maximum lateral accelerations and speed variance were not normally distributed. As the maximum lateral acceleration data was strongly positively skewed, logarithmic transformations were used for the analyses. The speed variance data was moderately positively skewed, and therefore square root transformations were applied based on the recommendations of Tabachnick and Fidell [34]. Analysis of variance (ANOVA) results are based on the transformed responses, while the graphs represent the original units. An alpha value of 0.5 was used as the criterion for statistical significance, and partial eta squared was used to measure effect sizes. Where Mauchly’s test indicated a violation of sphericity, degrees of freedom were Greenhouse-Geiser corrected.

## Results

### Response time

Although they were not explicitly instructed to do so, almost all drivers used their indicator in all three drives (N = 24). Therefore, drivers’ indication time was taken as the first signal of a decision to change lane. Response time was measured as the time from when the lead vehicle entered the driver’s lane to the time the indicator was first pressed.

**Fig 2:**
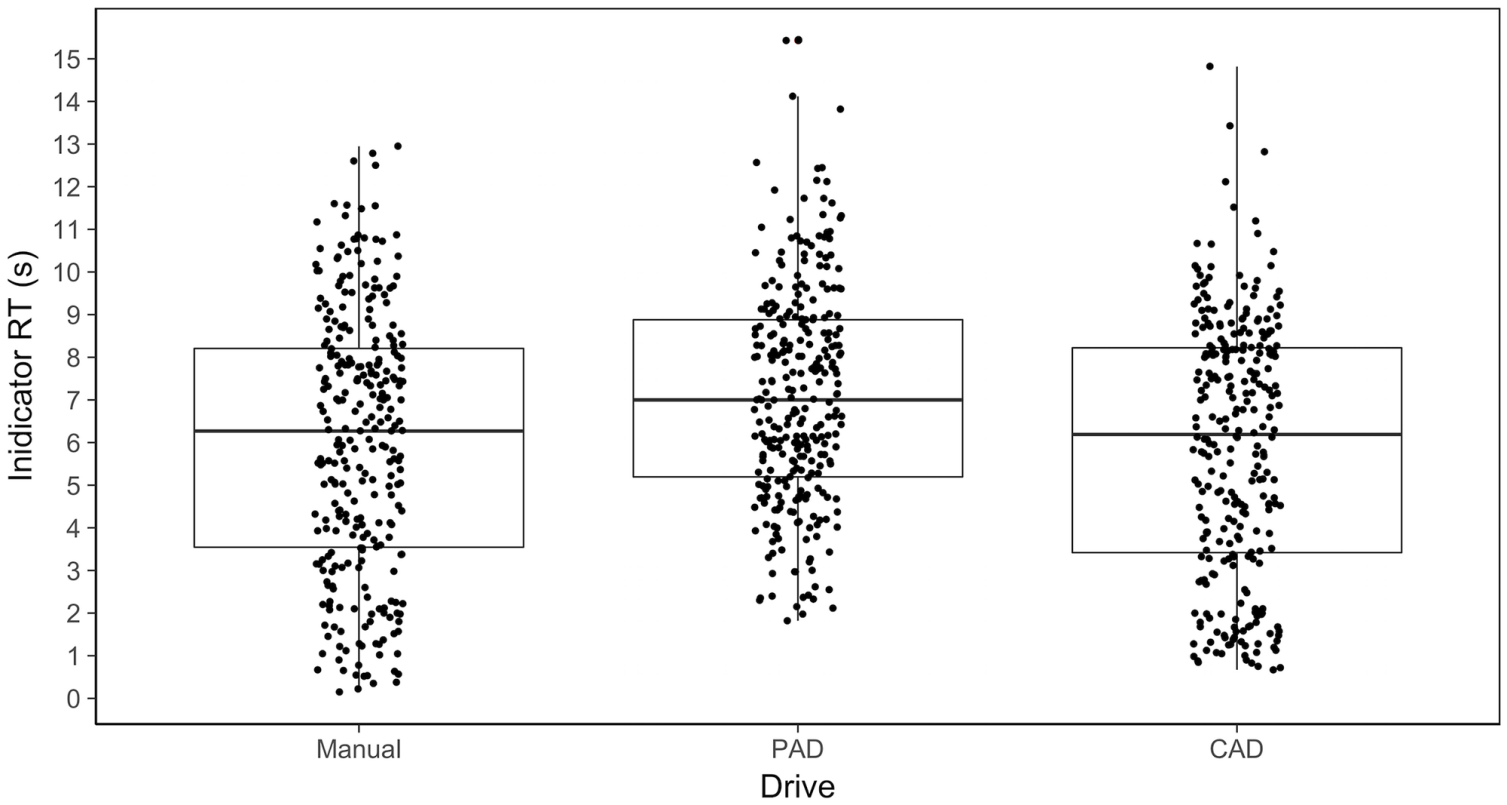
Comparing indicator response times across drives.

A 2-way repeated-measures ANOVA examining the effects of Drive (manual, PAD, and CAD), and Event (1–12) on indicator response times, showed a significant main effect of Drive (F(2,46) = 8.90, p<0.01, ηp^2^ = 0.28). As Fig 2 shows, participants took significantly longer to engage the indicator in PAD (M = 7.08s, SE = 0.44) than in either manual (M = 6.00s, SE = 0.55) or CAD (M = 5.90s, SE = 0.57). The extra time taken by drivers in PAD may have been needed for establishing situation awareness, perhaps by checking the system status and the surrounding traffic, before resuming control. There was no significant effect of Event (F(11,253) = 1.76, p = 0.06, ηp^2^ = 0.07), nor was there any interaction effect (F(9.04, 207.86) = 0.86, p = 0.65).

### Inverse time to collision and forward headway

Drivers use the looming retinal image of a lead vehicle as a cue for detecting its deceleration rate [35], and inverse time to collision (invTTC) provides a measure of this visual looming effect [17, 35–37]. To establish whether the looming effect of the lead vehicle had any effect on the time taken to initiate an overtaking manoeuvre, a 2-way repeated measures ANOVA examining invTTC at time of indication was calculated. The independent variables were Drive (manual, PAD, & CAD) and Event (1–12). There was no significant effect of Drive (F(2,56) = 1.92, p = 0.16) or Event (F(6.50,181.94) = 0.63, p = 0.80) on invTTC at indicator time. Therefore, it appears that the looming effect was not different in any of the three drives. The invTTC values ranged from 0.09 s^-1^ in CAD and PAD to 0.10 s^-1^ in manual, suggesting that drivers adopted a 10–11 second time to collision as a comfortable overtaking time in all three drives.

**Fig 3:**
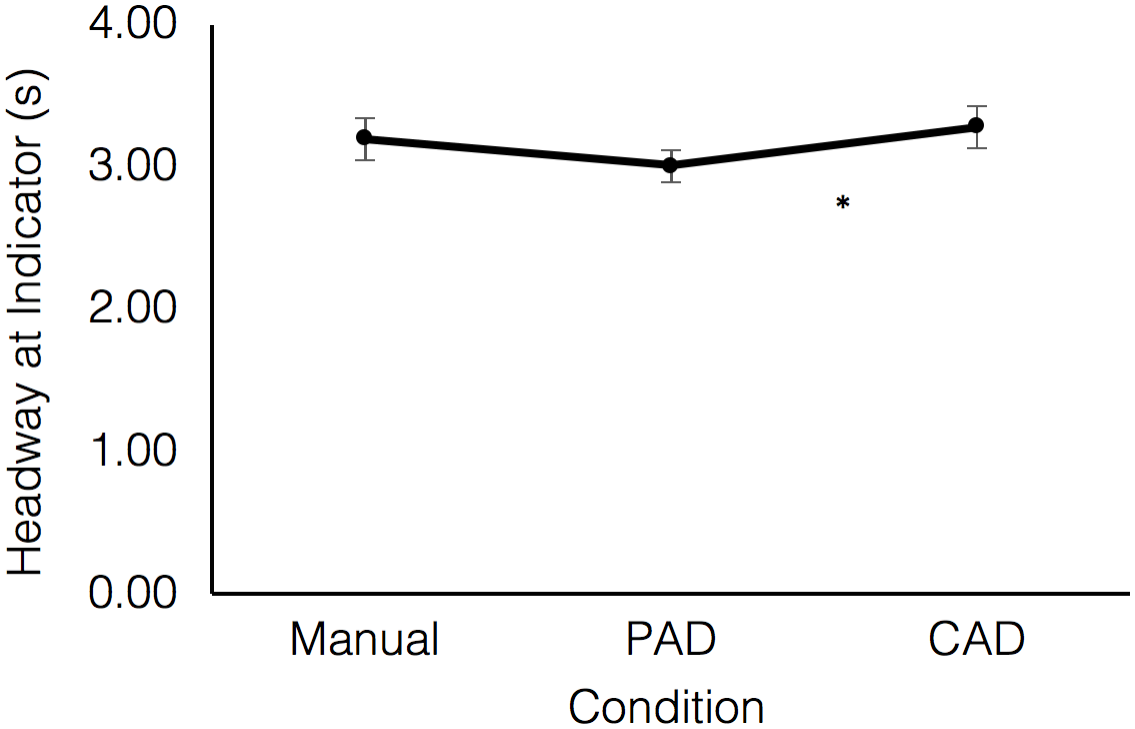
Forward headway in seconds at the point at which drivers used the indicator in all three drives. Error bars refer to SEM, *p<0.05.

To further explore the effects of the distance to the lead vehicle on overtaking manoeuvres, a 2-way repeated measures ANOVA was conducted with Time Headway (to the lead vehicle) at indicator time as the dependent variable. There was a significant effect of Drive (F(1.45, 33.26) = 7.44, p<0.01, ηp^2^ = 0.24), with pairwise comparisons showing that participants responded at a significantly shorter time headway in PAD (M = 2.98s, SE = 0.12) than in CAD (M = 3.29s, SE = 0.16; p<0.05; see Fig 3). There were no significant differences between manual driving (M = 3.16s, SE = 0.15) and either PAD (p = 0.14) or CAD (p = 0.06). There was no significant effect of Event (F(11, 253) = 1.62, p = 0.09, ηp^2^ = 0.07) on time headway, nor was there a significant interaction effect (F(8.59, 197.69) = 0.81, p = 0.71).

Taken together, these results imply that drivers in PAD were likely to take a little extra time to understand both the driving situation and how the system was working prior to initiating an overtaking manoeuvre. However, the fact that there was no significant differences in TTC across the groups suggests that the deceleration caused by the ACC ameliorated the relationship between speed and distance which would have increased the criticality of any looming effect.

### Vehicle control during manoeuvres

Numerous studies have explored lane changing trajectories during manual driving and automated driving under various conditions, for example as a result of driver distraction [38, 39], during visual occlusion [40, 41], and in different traffic densities [28, 42]. The following section uses some of the metrics identified in these studies to understand how PAD affected factors such as drivers’ lateral positioning, speed profile, and steering behaviour following a driver-initiated resumption of control in non-critical situations. As CAD did not require any vehicle control input from drivers, it is not included in the following analyses.

#### Automation disengagement method

**Fig 4:**
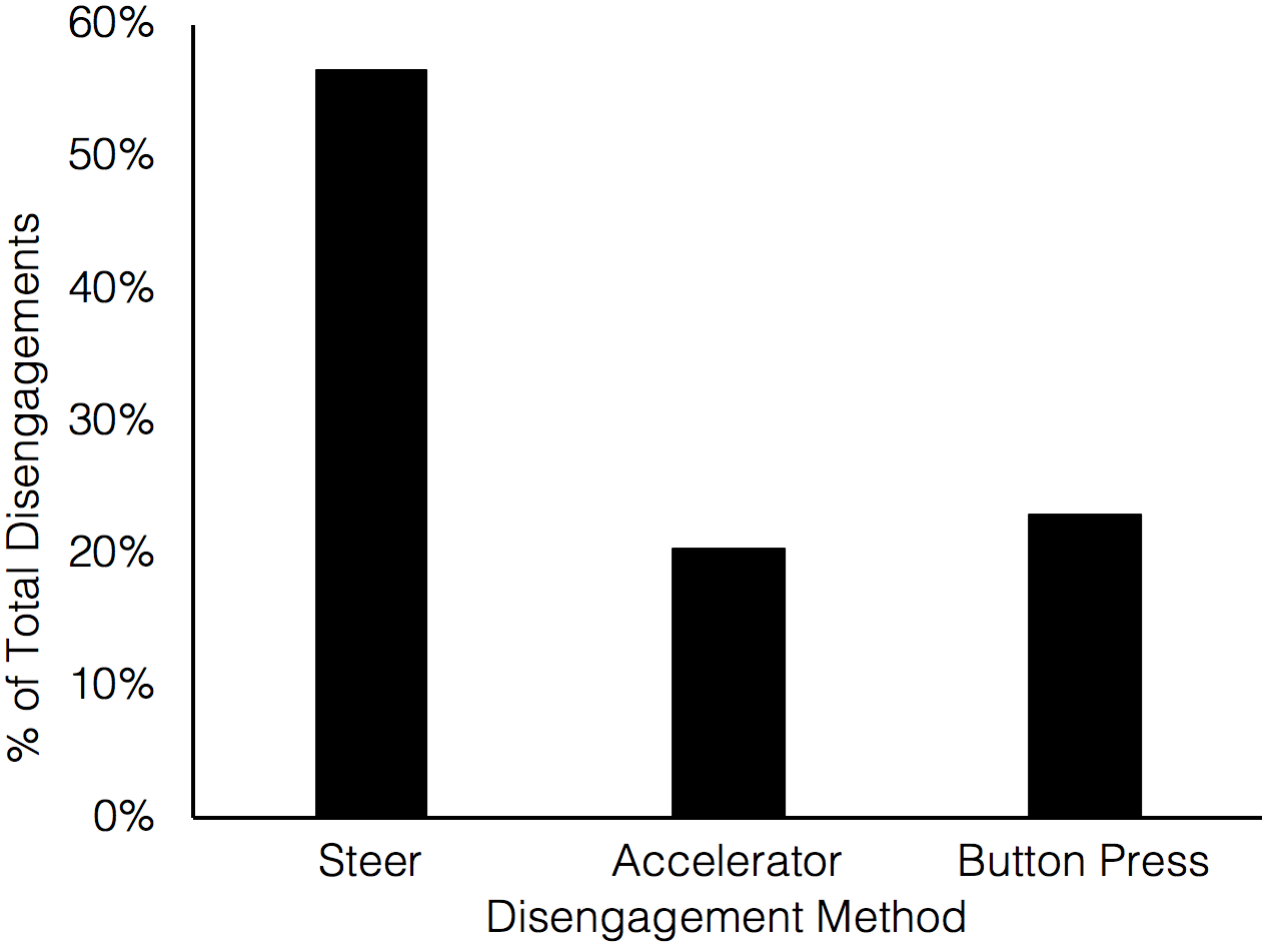
Disengagement methods used in PAD (percentage of total disengagements across participants and events).

In PAD, drivers could disengage automation by either pressing a button on the steering wheel, turning the steering wheel more than 2°, or pressing the accelerator or brake pedals. As shown in Fig 4, the majority of disengagements occurred by turning the steering wheel, followed by button press disengagements, and use of the accelerator pedal. This is perhaps unsurprising as the lane-change manoeuvre required participants to use the steering wheel to change their trajectory. The brake pedal was not used as a disengagement tool by any participant in this experiment.

#### Lateral position

The standard deviation of lateral position (SDLP) relative to the centre of the road was used to provide a measure of the quality of the steering movement during the overtaking manoeuvre [21, 39]. A two-way ANOVA was conducted to examine the effect of Drive and Event on SDLP. As all drivers completed their overtaking manoeuvre at a different time and position along the road, the start of each driver’s overtaking trajectory was anchored around the point at which the lead vehicle appeared in their lane, and measured for 40 seconds after this point. This time window was sufficient to ensure that all lane changes were captured.

**Fig 5:**
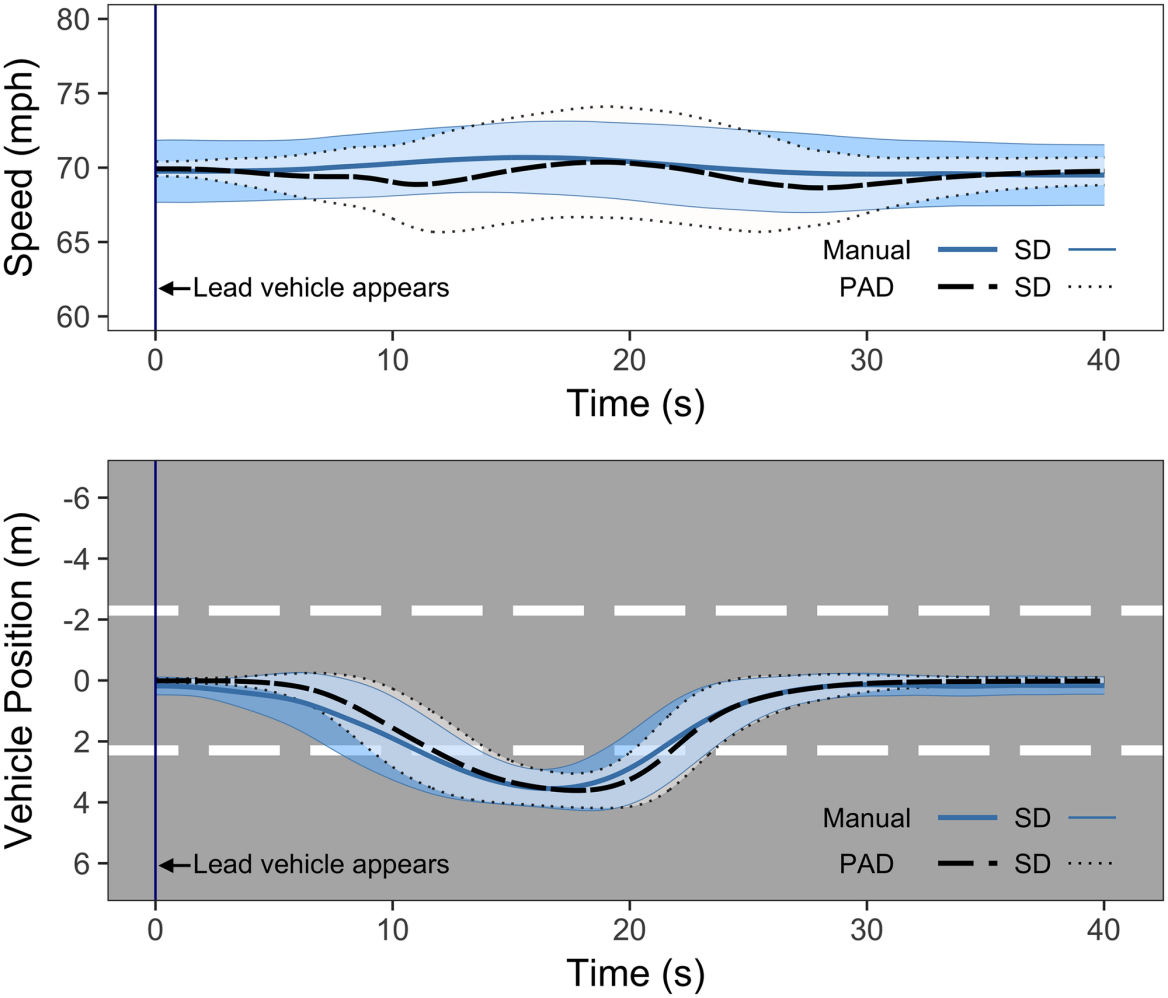
Drivers’ lateral position (top) and speed (bottom) during lane changes. The lines represent mean position, with the dark shaded area representing standard deviation in manual driving, and the light shaded area representing standard deviation in PAD.

Results indicate that SDLP was significantly larger in PAD (M = 1.45m, SE = 0.03) than in manual driving (M = 1.39m, SE = 0.03; F(1,28) = 13.31, p<0.01, ηp^2^ = 0.32; see Fig 5). There was no significant effect of Event (F(5.53, 154.93) = 1.57, p = 0.11) and no interaction effect (F(6.39, 178.97) = 0.46, p = 0.93). As shown in the top graph of Fig 5, drivers started the manoeuvre later in PAD and had a slightly sharper trajectory than in manual driving, confirming the earlier analyses of indicator response time and time headway.

#### Speed profiles

In order to compare speed behaviour during manual and PAD, drivers’ speed profiles were also anchored around the lead vehicle appearance and measured for 40 seconds after this point. A two-way repeated measures ANOVA on mean speed during this time showed no significant effect of Drive (F(1, 28) = 2.37, p = 0.14) or Event (F(5.27, 147.58) = 0.81, p = 0.63) across the 24 manoeuvres (manual and PAD). However, a second two-way ANOVA on the standard deviation of speed during the overtaking manoeuvre found that speed variance was significantly higher in PAD, compared to manual driving (F(1,28) = 49.63, p<0.001, ηp^2^ = 0.64). The bottom graph in Fig 5 shows that this variance lasted across the overtaking manoeuvre, suggesting that drivers were less consistent in maintaining their speed after resuming control from automation. These results suggest that the process of turning off the automated system, and resuming control of the brake and accelerator pedals led to fluctuations in speed as drivers became accustomed to the force required to control the vehicle. The speed instability remained across the 12 overtaking manoeuvres, suggesting that the destabilising effects of resuming control from automation did not reduce with repeated exposures. There was no significant effect of Event on the standard deviation of speed (F(11,308) = 1.09, p = 0.37), nor was there any interaction effect (F(11, 308) = 1.07, p = 0.39).

#### Lateral acceleration

To further explore drivers’ vehicle control during the overtaking manoeuvre, maximum lateral acceleration in manual driving and PAD were compared. This measure is considered to be a good indicator of the level of sharpness or jerkiness associated with a lane change [16].

As the overtaking manoeuvre involved changing lanes in two different directions (into and out of the third lane), the metrics for exiting and re-entering the lane were considered separately for this analysis. Previous studies have shown that steering wheel movements during a lane change consist of three sub-movements, the first of which usually provides the greatest change in positioning and the sharpest movement [21, 43]. We expected that this movement would occur prior to the point at which the greatest deviation in road position occurred. Thus, the maximum lateral acceleration for the lane exit was measured from the point at which the lead vehicle appeared to the point at which the greatest deviation in road position to the right occurred. The maximum lateral acceleration for lane re-entry was measured from this point to the point at which the greatest deviation in road positioning to the left was achieved.

**Fig 6:**
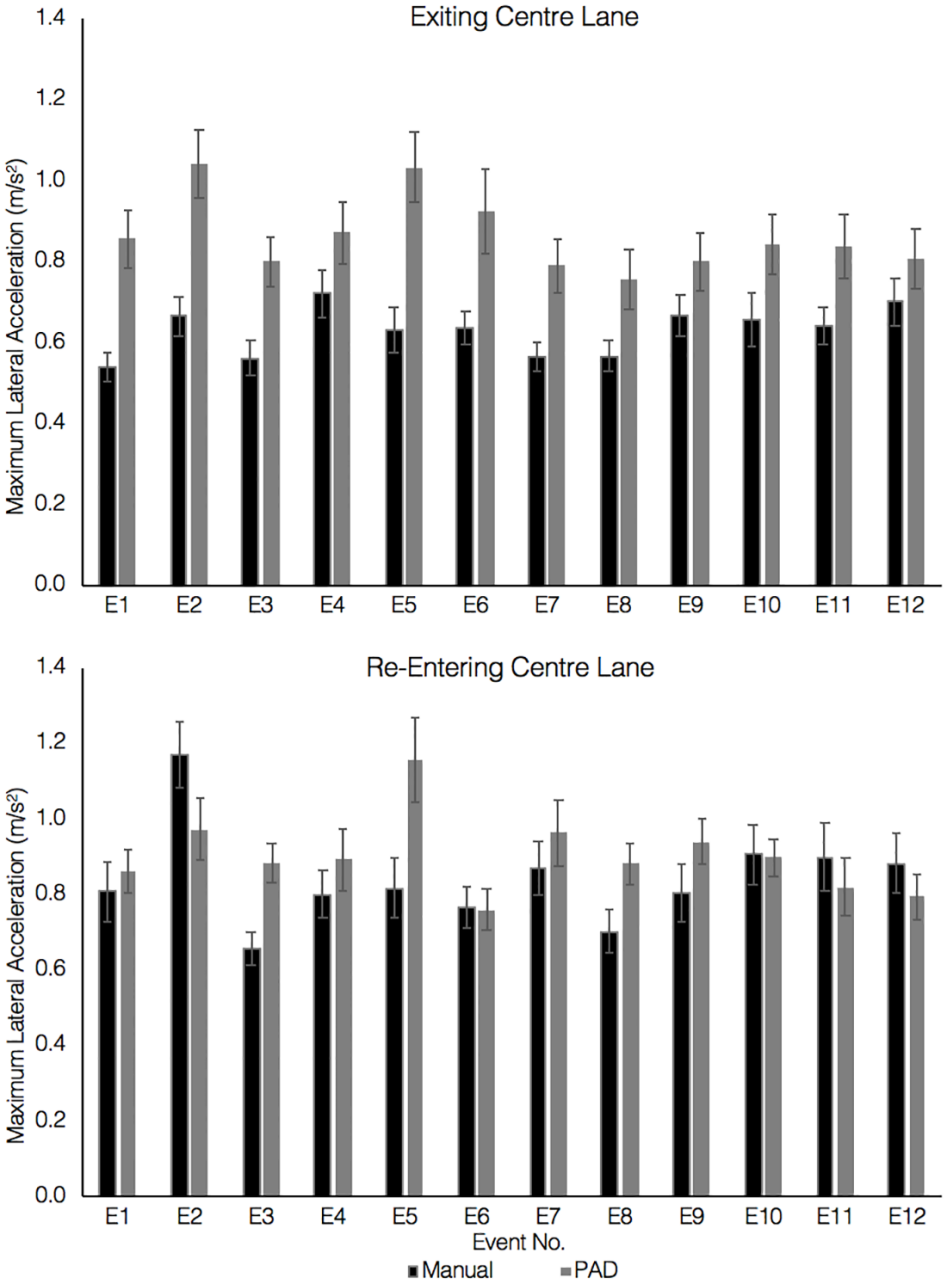
Three way interaction between drive, event and direction on lateral acceleration. Error bars refer to SEM.

A three-way repeated measures ANOVA was conducted on drivers’ maximum lateral acceleration, with Drive (manual and PAD), Event (1–12), and Direction (lane exit, lane re-entry) as the independent variables. Results indicate a significant main effect of Drive on maximum lateral acceleration during the overtaking manoeuvres (F(1,28) = 46.39, p<0.001, ηp^2^ = 0.62) with drivers having higher lateral accelerations following the use of the PAD system (M = 0.88m/s^2^, SE = 0.04) than in manual driving (M = 0.73m/s^2^, SE = 0.03). There was also a significant effect of Event (F(11,308) = 5.04, p<0.001, ηp^2^ = 0.15). Post-hoc pairwise comparisons revealed that this was the result of a significant increase in maximum lateral accelerations at Event(E) 2 (M = 0.96, SE = 0.05), compared to E1 (M = 0.76, SE = 0.04), E3 (M = 0.72, SE = 0.04), E8 (M = 0.73, SE = 0.04), and E12 (M = 0.79, SE = 0.04). There were no other significant differences between the events. An examination of Fig 6 suggests that there were higher accelerations for PAD during lane exit at Event 2, but for manual driving there were higher accelerations during lane re-entry at Event 2. The main effect of Event number incorporates both of these elements, suggesting that the effect was a result of drivers’ becoming accustomed to the vehicle handling required for the task. There was also a significant effect of Direction (F(1,28) = 26.22, p<0.001, ηp^2^ = 0.48), with drivers having lower maximum lateral accelerations when exiting the centre lane (M = 0.75, SE = 0.04) than when re-entering (M = 0.87, SE = 0.04).

There were a number of significant interaction effects. Firstly, there was a significant interaction between Drive and Event (F(6.72,188.13) = 2.94, p<0.01, ηp^2^ = 0.10). There was a reduction in maximum lateral accelerations across events in PAD, which led to a decrease in the differences between PAD and manual driving. There was also a significant interaction between Drive and Direction (F(1,28) = 21.89, p<0.001, ηp^2^ = 0.44), with a much larger difference in maximum lateral accelerations between PAD and manual driving during lane exit than lane re-entry. Finally, there was a significant three-way interaction between Drive, Event, and Direction (F(6.81,190.57) = 2.43, p<0.01, ηp^2^ = 0.08), which is displayed in Fig 6. The largest differences in maximum lateral accelerations between manual driving and PAD occurred while moving into the overtaking lane during the first six events. The size of the Drive differences diminished across the final 6 events, suggesting that drivers had learned to re-take control more smoothly after around the sixth event. However, the lack of overlap between the error bars shows that lateral accelerations during PAD were still significantly higher than in manual driving. On re-entry to the centre lane after overtaking, the difference in maximum lateral acceleration between manual and PAD was smaller, suggesting that drivers’ vehicle control in PAD had become more stable over the time taken to complete the overtaking manoeuvre. Nevertheless, there was still a sizeable difference for the majority of drivers during the first 5 events. The maximum lateral acceleration values were higher for both manual driving and PAD when re-entering the centre lane, suggesting that regardless of condition, drivers moved sharply back into the middle lane once they had overtaken the slow-moving vehicle.

### Subjective evaluation

The final analyses focused on gaining an understanding of drivers’ subjective evaluations of both of the vehicle automation systems. In this paper, we focused on drivers’ evaluations based on two different questions. At the end of the experiment, drivers were asked to select their preferred automated system—CAD or PAD. The majority of drivers (60%) preferred the conditionally automated system to the partially automated one (36.7%), with 3.3% participants failing to select a favourite.

**Fig 7:**
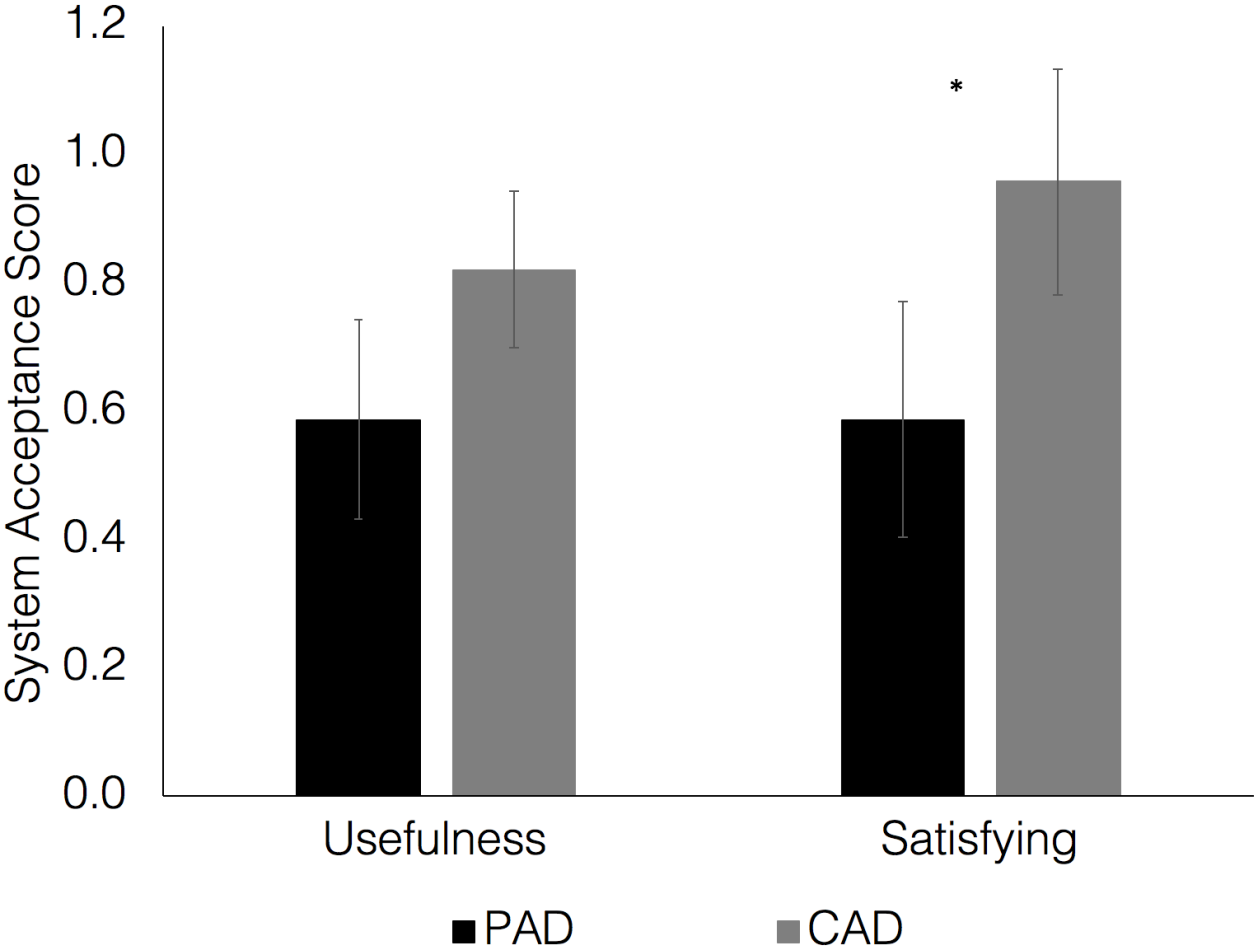
Drivers’ acceptance ratings for PAD and CAD. Error bars refer to SEM.

Drivers were also asked to provide ratings of system acceptance using Van der Laan et al.’s [31] scale, comprising of items measuring how useful and satisfying users found each system. Results showed that there were no significant differences in the ratings of system usefulness (t(28) = 2.03, p = 0.05). However, participants rated the CAD system as being significantly more satisfying to use (t(28) = 2.63, p<0.05; see Fig 7).

## Discussion

Although there is increasing evidence to suggest that vehicle automation leads to performance decrements during transfers of control in critical situations [10, 11, 13, 16], there has been little investigation of the quality of driver-initiated transfers in non-urgent situations. This is an important issue, as users of SAE Level 2 and Level 3 vehicle automation are likely to encounter these types of non-urgent situations on a regular basis, for example, if they wish to change lane during motorway driving. Therefore, the aim of the current study was to address two main gaps in the literature. To begin, the study provides one of the first investigations into the vehicle control implications of driver-initiated transitions from automation. Secondly, the study provides a comparison of the effects of different levels of automation on drivers’ vehicle control in situations which are not time-critical, by comparing how and when they initiated an overtaking manoeuvre in manual driving, PAD, and CAD.

As outlined in the Introduction, the study specifically addressed four main questions. Our first two questions investigated whether there were any differences between manual driving, PAD, and CAD, regarding the time taken by drivers to initiate an overtaking manoeuvre, and the distance to the lead vehicle at which they initiated this manoeuvre. Eriksson & Stanton [20] showed that when drivers were given a takeover request without a time restriction, transition times were substantially longer than those reported in time-critical studies. However, the transitions in their study were initiated by a system reminder, and were not linked to any changes in the driving environment. Our results show that when asked to respond to elements in the environment i.e. a slow moving lead vehicle, drivers had slightly longer response times in PAD than in manual driving or CAD, and got closer to the lead vehicle before initiating a lane-change. This provides some support for the idea that drivers will take additional time, when available, to regain an understanding of the situation before re-entering the vehicle control loop. However, on average this process only took one extra second, and may just have been a result of drivers moving their hands and feet into position for driving, or checking the system to see who was in control. This implies that even in non-urgent situations, where the ACC would protect them from a crash, drivers do not take much time to re-orient themselves to the situation prior to taking control from automation. There were no significant differences between the inverse TTC values at indicator time, suggesting that the looming effects were the same in all three drives. For the automated drives, the ACC adapted drivers’ speed to maintain a minimum time headway of 2 seconds. As drivers initiated their overtaking manoeuvres at approximately a 3 second headway, it is likely that the ACC had started to decelerate, thus minimising the effects on TTC of any slight variations in headway.

Our third question was to establish whether there were any differences in the quality of the overtaking manoeuvre during manual driving compared to during the resumption of control from PAD. Our results provide evidence that even in driver-initiated transfers, with low criticality, there are still significant differences in vehicle control between manual driving and PAD. Drivers displayed greater fluctuations in their speed and lateral position when re-taking control from automation. It is possible that this is a function of the way in which automation was de-activated. For example, if drivers de-activated using the steering wheel, the very action of having to turn the steering wheel more than 2 degrees to turn off automation may have contributed to a sharp trajectory for some drivers. Thus, it may be that this method of disengagement should be avoided when vehicle manufacturers are designing their disengagement criteria. In addition, the process of transferring control of the brake and accelerator pedals is likely to lead to fluctuations in speed while drivers become accustomed to the force required for normal vehicle control. Merat et al. [13] found that it took drivers 35–40 seconds to stabilise their lateral vehicle control after a transfer from automation. The entire overtaking manoeuvre in the current study took less than 30 seconds, suggesting that during a simple overtaking manoeuvre there is not sufficient time for adequate vehicle stabilisation. Interestingly, it appears that increased exposure improved drivers’ ability to control some elements of the transition, with an examination of maximum lateral accelerations showing that the difference between manual and PAD reduced during the final six events when the maximum lateral accelerations in PAD became more consistent. This builds on previous research with both ACC and higher levels of automation, which shows that drivers who are familiar with a system are more likely to respond appropriately [15, 44, 45]. However, although the ability to control the vehicle after a transition improved over time, at least regarding lateral accelerations, responses were still higher in PAD than in manual, suggesting that the learning effect cannot fully mitigate the detrimental effects of being out of the loop during the transfer of control. This variability in speed and vehicle positioning could have the potential to cause confusion for other traffic, and may lead to dangerous interactions if there are other vehicles travelling in the overtaking lane.

Our final question was to evaluate whether there were any differences in drivers’ evaluations of using different levels of driving automation. A number of authors suggest that automated driving systems should attempt to mirror individuals’ driving styles to increase acceptance and use of these systems (e.g. [46]). However, although drivers enjoyed using both automated systems, they preferred the CAD system, even though its lane-change trajectory was quite different from that adopted by drivers in manual and PAD. This suggests that, given a choice, drivers prefer not to have to intervene with the automated system, even when not engaging in other tasks. In addition, the requirement for automated systems to mirror an individual’s driving style may be less important than previously suggested, a finding supported by a two recent studies which showed that drivers did not necessarily prefer an automated system that matched their driving style [30, 47]. These findings have implications for the potential success of endeavours to decrease vehicle emissions and improve traffic flow through increased vehicle automation and electrification [48, 49]. If drivers are happy to use an automated system which doesn’t match their driving style, then they are more likely to accept a vehicle which adopts a slower speed or smoother trajectory than they would when driving themselves.

As with all studies, there are some limitations which must be acknowledged. The current study required drivers to overtake 12 times per drive, with each overtaking event occurring in very similar circumstances. The repetitive nature of the task is likely to have impacted on their behaviour, which may have been more varied if the conditions surrounding the overtaking process were changeable. In addition, there was never any traffic in the overtaking lane, meaning the lead vehicle was the only element of the road environment to influence drivers’ responses. Additional research is needed to understand if the same responses would be made if drivers also needed to consider the size of the gap available in the overtaking lane. It would also be interesting to understand whether drivers would choose to overtake at all if not instructed to do so.

### Conclusions

The current study compares drivers’ overtaking behaviour in manual driving, PAD, and CAD; providing insights into chosen headways and vehicle control capabilities in non-urgent situations. Drivers appeared to enjoy using both PAD and CAD systems, suggesting that acceptance of these systems is likely to be high, at least as long as there are no system failures.

Previous research has tended to focus on the effects of vehicle automation during system-initiated transfers of control in critical situations. By focusing on non-urgent, driver-initiated, transfers of control, the results of this study provide an important contribution to our understanding of the impacts of different levels of automation on driving performance. The vehicle control metrics indicate that even in non-urgent situations, there are safety implications of re-taking control from vehicle automation, which must be considered when designing these systems. Our results show that the additional second taken by drivers to initiate a lane change in PAD was not sufficient for them to regain full situation awareness, with increased variability in vehicle positioning, and both longitudinal and lateral speed remaining an issue throughout the overtaking manoeuvre. This suggests that even when a driver has control of when to re-enter the driving loop, the effects of being out-of-the-loop remain, which has implications for vehicle manufacturers designing for transitions of control. The results highlight the importance of considering the most effective disengagement criteria, and emphasize the possible difficulties associated with SAE Level 2 and Level 3 systems, which will require drivers to re-enter the driving loop occasionally. Further research is required to understand if solutions such as providing more informative HMI or shared haptic control [50, 51], or solutions which imbed the automated vehicle technology within smart infrastructure [52], would enable a smoother and safer transfer of control in these situations.
